# Feasibility Study for Multimodal Image-Based Assessment of Patient-Specific Intracranial Arteriovenous Malformation Hemodynamics

**DOI:** 10.3390/jcm14082638

**Published:** 2025-04-11

**Authors:** Janneck Stahl, Laura Stone McGuire, Tatiana Abou-Mrad, Sylvia Saalfeld, Daniel Behme, Ali Alaraj, Philipp Berg

**Affiliations:** 1 Research Campus STIMULATE, University of Magdeburg, 39106 Magdeburg, Germany; saalfeldlab@gmail.com (S.S.); daniel.behme@med.ovgu.de (D.B.); philipp.berg@ovgu.de (P.B.); 2 Department of Medical Engineering, University of Magdeburg, 39106 Magdeburg, Germany; 3 Department of Neurological Surgery, University of Wisconsin-Madison, Madison, WI 53792, USA; mcguire@neurosurgery.wisc.edu; 4 Department of Neurosurgery, University of Illinois, Chicago, IL 60612, USA; tamrad@uic.edu (T.A.-M.); alaraj@uic.edu (A.A.); 5 Institute for Medical Informatics and Statistics, University Hospital Schleswig-Holstein, Campus Kiel, 24105 Kiel, Germany; 6 Department of Neuroradiology, University Clinic of Magdeburg, 39120 Magdeburg, Germany

**Keywords:** arteriovenous malformations, computational fluid dynamics, hemodynamics, image segmentation, multimodal imaging

## Abstract

**Background/Objectives**: Intracranial arteriovenous malformations (AVMs) exhibit a complex vasculature characterized by a locally occurring tangled nidus connecting the arterial and venous system bypassing the capillary network. Clinically available imaging modalities may not give sufficient spatial or temporal resolution. Adequate 3D models of large vascular areas and a detailed blood flow analysis of the nidus including the surrounding vessels are not available yet. **Methods**: Three representative AVM cases containing multimodal image data (3D rotational angiography, magnetic resonance angiography, magnetic resonance venography, and phase-contrast quantitative magnetic resonance imaging) are investigated. Image segmentation results in partial 3D models of the different vascular segments, which are merged into large-scale neurovascular models. Subsequently, image-based blood flow simulations are conducted based on the segmented models using patient-specific flow measurements as boundary conditions. **Results**: The segmentation results provide comprehensive 3D models of the overall arteriovenous morphology including realistic nidus vessels. The qualitative results of the hemodynamic simulations show realistic flow behavior in the complex vasculature. Feeding arteries exhibit increased wall shear stress (WSS) and higher flow velocities in two cases compared to contralateral vessels. In addition, feeding arteries are exposed to higher overall WSS with increased value variation between individual vessels (20.1 Pa ± 17.3 Pa) compared to the draining veins having a 62% lower WSS (8.9 Pa ± 5.9 Pa). Blood flow distribution is dragged towards the dominating circulation side feeding the nidus for all the cases quantified by the volume flow direction changes in the posterior communicating arteries. **Conclusions**: This multimodal study demonstrates the feasibility of the presented workflow to acquire detailed blood flow predictions in large-scale AVM models based on complex image data. The hemodynamic models serve as a base for endovascular treatment modeling influencing flow patterns in distally located vasculatures.

## 1. Introduction

Intracranial arteriovenous malformations (AVMs) are, in general, inborn neurovascular pathologies directly connecting arterial and venous vasculature via a tangle-like nidus. The nidus is supplied by arterial feeders and drainage to sinuses is conducted via draining veins. With a rather low prevalence of 18 per 100,000 in the Western population [1], AVMs possess a hemorrhage risk of 2 to 3% per year [2,3]. Bleedings are associated with high rates of morbidity and mortality. Until now, the mechanisms leading to AVM bleeding are poorly understood. Nevertheless, critical hemodynamic factors such as increased flow resulting in higher shear rates and high arterial or venous pressure are important factors contributing to AVM hemorrhage [4,5].

In the last decade, computer-assisted methods based on image processing and computational fluid dynamics (CFD) have been involved in assessing the stability of neurovascular diseases and simulating the therapy impact of several treatment devices [6,7,8]. However, those studies consider rather small regions of interest, whereby the surrounding vasculature in particular can have a considerable influence on the local blood flow in these pathologies [9]. Nevertheless, a sustained innovation drive occurred in recent years towards the venous side [10,11]. Since brain AVMs affect the entire vascular system, it is of special interest to obtain a broad overview of this disease with respect to both morphology and blood flow. Prior studies already investigated various segmentation methods extracting patient-specific AVM morphologies based on medical image data. Colombo et al. [12] reviewed 33 studies reporting 3D segmentation techniques for AVMs, concluding that there is no gold standard for segmenting AVMs yet, in particular for the assessment of hemodynamics. Numerical studies related to AVM hemodynamics are limited in simplifying the vessel geometries to biomathematical networks [13] or with a porous medium representing the nidus [14]. Kaneko et al. [15] were able to extract complex nidus vessels for conducting blood flow simulations. However, the segmented region of interest is still small compared to the impact AVMs have on the large arteriovenous vasculature.

The proposed study addresses these limitations by introducing an advanced workflow using multimodal medical image data for generating 4D AVM models including large vascular domains and realistic representations of the nidus. Based on comprehensive image-processing steps image-based blood flow simulations using patient-specific flow measurements are conducted. The purpose of this study is to assess the hemodynamic impact of three AVM pathologies by incorporating extensive vascular regions, enabling the evaluation of blood flow alterations in the upstream arteries and downstream veins surrounding the nidus. Specifically, it seeks to quantify the biomechanical stresses induced by the AVM nidus to enhance the understanding of AVM-related hemodynamics and provide a foundation for more informed clinical decision making.

## 2. Materials and Methods

**Figure 1:**
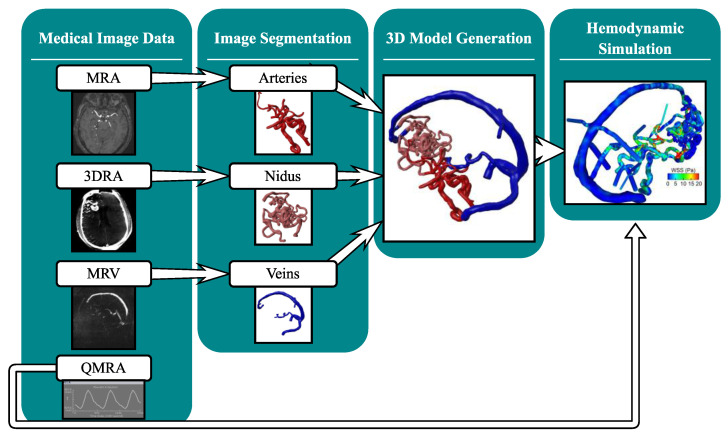
Developed workflow for the processing of multimodal medical image data (magnetic resonance angiography: MRA, 3D rotational angiography: 3DRA, magnetic resonance venography: MRV, and phase-contrast quantitative magnetic resonance imaging: QMRA) for the hemodynamic investigation of large-scale AVM models.

This study presents a pipeline to conduct comprehensive 4D modeling including image-based blood flow simulations on patient-specific AVM image data. Figure 1 depicts the investigated workflow described in detail in this feasibility study.

### 2.1. Patient and Image Data

**Figure 2:**
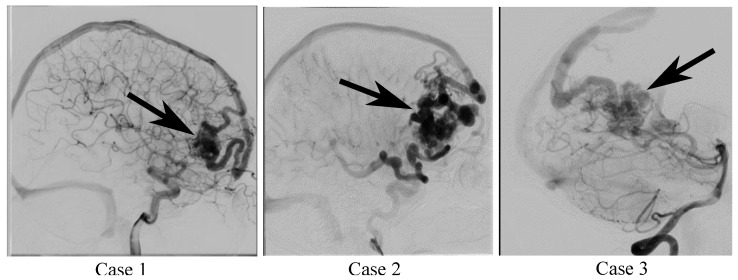
Planar angiographic images from the sagittal view of the three AVM cases. The imaging sequences show the filling of the nidus and partly of the local sinuses. The black arrows indicate the nidus location.

Three AVM cases were selected to accommodate the individual patient-specific variability. They were acquired with various imaging modalities and differ in their vascular morphology and size as well as the location of the nidus (see Figure 2). Two AVM nidus were supplied by the anterior circulation (cases 2 and 3) and one nidus mainly by the posterior circulation (case 3).

For all three cases, four different modalities of image data were available: (1) Magnetic resonance angiography (MRA) data were acquired to resolve the arterial vessels. Here, both circulations—anterior and posterior—could be resolved. (2) The 3D rotational angiography (3DRA) was available to resolve the nidus and the feeding and draining vessels. (3) The acquisition of the venous vasculature was conducted using magnetic resonance venography (MRV) showing the draining veins and the sinus. (4) Phase-contrast quantitative magnetic resonance imaging (QMRA) was acquired to quantify the vascular flow using non-invasive optimized vessel analysis (NOVA, VasSol, Inc., River Forest, IL, USA). Here, the flow was captured perpendicular to the relevant vessel axis. These measurements were available at almost all the major cerebral arteries and veins serving as patient-specific boundary conditions for the hemodynamic simulations. Table 1 summarizes the morphologic characteristics of the three acquired AVM cases based on the image data.

### 2.2. Multimodal Image Segmentation and 3D Model Generation

Based on the multimodal DICOM images of the AVMs, the image processing tool collection MeVisLab v3.4.3 (MeVis Medical Solutions AG, Bremen, Germany) was used to extract the complex vasculature. Before segmenting the AVM vasculature, the image data were translated into one joint coordination system. This was achieved by a 3D image-based rigid body registration pre-implemented in MeVisLab.

For the initial image segmentation, threshold-based methods were applied to the contrast-enhanced image data for creating vessel segmentation masks. The applied lower threshold was adjusted empirically according to comparisons to the planar angiographic data (see Figure 2) by medical engineers with more than ten years of experience related to vascular image processing. The cross-referencing with 2D angiographic data enabled the use of the highest available spatial resolution. In addition, this method has proven effective in the systematic analysis within the MATCH study [16], particularly when assessing segmentation accuracy in a quantitative manner. The arterial branches including the major vessels of the Circle of Willis were segmented based on the MRA data. Based on the 3DRA data, the nidus and its surrounding feeding and draining vessels were extracted, since these images contain the highest spatial resolution, especially for the narrow vascular courses inside this structure. The venous system starting from the draining veins towards the deep sinus vessels was segmented based on the MRV images.

After the segmentation process, three partial segmentation masks were available for each AVM case. These different vascular structures were converted into triangulated 3D surface meshes using the Marching Cubes algorithm available within MeVisLab. To exclude mesh parts, which do not belong to the AVM vasculature, a connected component analysis was conducted. Since the segmented vascular structures contained mesh artifacts due to the limited resolution of the imaging systems compared to the vessel diameters, manual corrections were conducted using the open-source 3D modeling suite Blender v3.0 (Blender Foundations, Amsterdam, The Netherlands). These post-processing steps contained the separation of incorrectly fused vessel regions and the removal of further surface mesh artifacts. In addition, the vessels were smoothed to minimize the influence of artificial aspects on the subsequently simulated blood flow.

**Figure 3:**
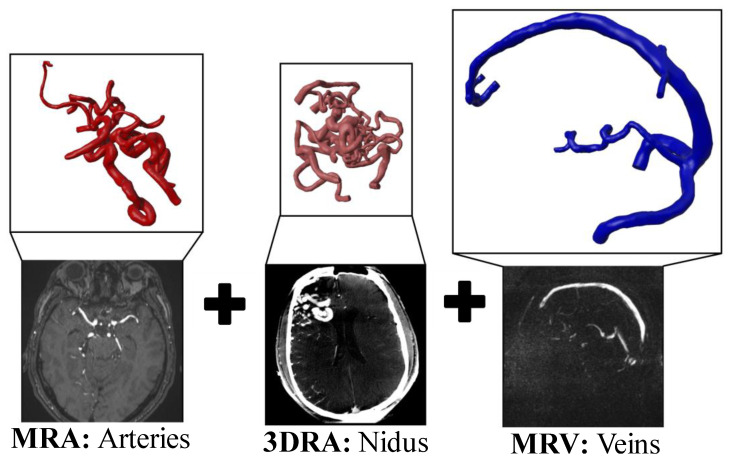
Extracted individual vessel parts of the AVM case 2 including the underlying imaging modalities. The MRA data result in arterial vessel models (**left**), 3DRA in nidus vessel models (**middle**), and MRV yielding sinus vasculature (**right**).

Figure 3 shows the post-processed partial vascular segmentations of one representative case based on the different imaging modalities. The individual models of the AVM morphology are connected using Boolean operations resulting in an overall AVM model including the arterial and venous system. To prepare the models for hemodynamic simulations, the corresponding inlets and outlets were cut perpendicular to the vessel axis and extruded by at least six times the length of the corresponding vessel diameter [17,18]. This ensured the complete development of the velocity profile from the inlet and reduced reverse flows at the outlets.

### 2.3. Hemodynamic Simulation

The numerical blood flow simulations were carried out based on CFD. Here, the finite-volume-based solver STAR-CCM+ v17.06 (Siemens Product Lifecycle Management Software Inc., Plato, TX, USA) was used to solve the equations of conservation of mass and momentum. To prepare the AVM models for the hemodynamic simulations, spatial discretization was carried out. Volume meshes were generated from the segmented 3D surface models. In particular, polyhedral cells with a base size of 0.25 mm were used. Furthermore, to account for the velocity gradients arising especially due to the narrow and tangled vessels, three prism layers with a growth rate of 1.3 were generated on the luminal surface. These settings resulted in a total number of elements of 3.2 million for case 1, 8.1 million for case 2, and 4.5 million for case 3, respectively, which were found to be adequate according to a preliminary mesh sensitivity analysis [19]. Aiming at a realistic hemodynamic prediction, patient-specific flow values were applied as boundary conditions using the QMRA flow measurements for each case. For all major arteries and veins, these patient-specific flow values were defined as in- and outflow conditions. However, for the limited number of smaller side branches at the venous side, representative pressure and velocity values were taken from the literature [20,21]. In all three cases, rigid and no-slip wall conditions were applied to the vessel walls since no further information could be extracted from the clinical image data. To ensure a subsequent comparability of the results, incompressible $\left(\rho =1055\;kg/m^{3}\right)$ and Newtonian $\left(\eta =0.004\;Pa\;s\right)$ fluid properties were defined for blood. In addition, a laminar flow behavior was assumed.

### 2.4. Analysis of Hemodynamic Parameter

**Figure 4:**
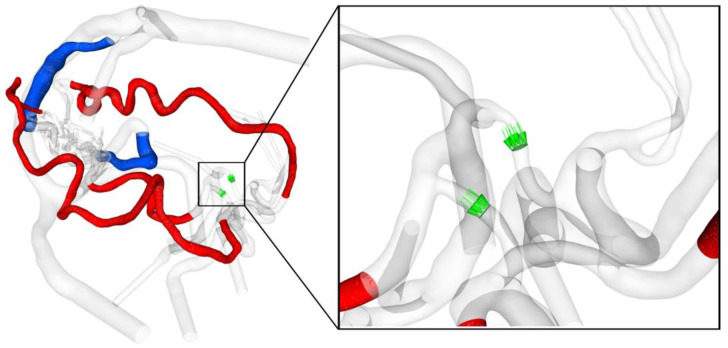
Visualization of the feeding arteries in red and the draining veins in blue, where the wall shear stress is quantified. In the magnification on the right, the cross-sectional planes and the direction of the volume flow rate quantification from posterior to anterior are shown in green.

After exporting the simulated data as binary encoded EnSight Gold files, post-processing and flow visualization were performed using EnSight v10.2.8 (ANSYS Inc., Canonsburg, PA, USA). In addition to occurring velocity patterns, this study elaborated on the wall shear stress (WSS) describing the tangential stress of the blood that is applied to the lumen of the vessel walls [22]. Quantifications were made along the entire course of the feeding arteries and draining veins. Moreover, the volume flow rate across the posterior communicating arteries was calculated. For all the cases, this was considered from the posterior to the anterior direction to analyze the flow direction (see Figure 4).

## 3. Results

### 3.1. Three-Dimensional Modeling Results

**Figure 5:**
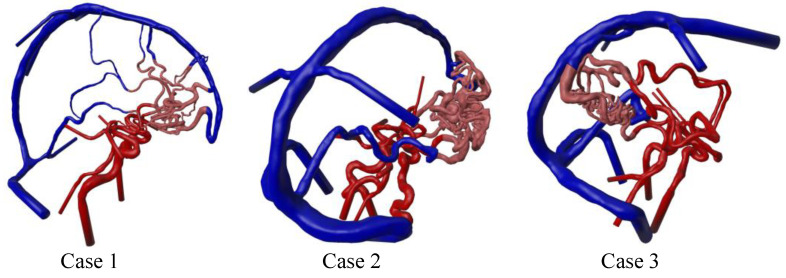
Segmented and post-processed 3D surface models of the three AVM cases with colored arteries (red), nidus (light red), and veins (blue). The inlet and outlet surfaces are extruded to ensure stability within the numerical simulations.

Based on the comprehensive image processing pipeline, large-scale AVM 3D models were achieved for all three cases (see Figure 5). The preserved models cover the arteriovenous area from the anterior and posterior Circle of Willis circulation to the transverse sinus veins. All the feeding arteries and draining veins are shown in their entire course. The nidus vessels could be separated and demonstrate corresponding vessel diameters resolvable by the underlying 3DRA imaging data. Due to the rigid-body co-registration of the multimodal image data, all the vessel models are correctly aligned for the fusion process.

### 3.2. Hemodynamic Investigation of Shear-Related Phenomena

**Figure 6:**
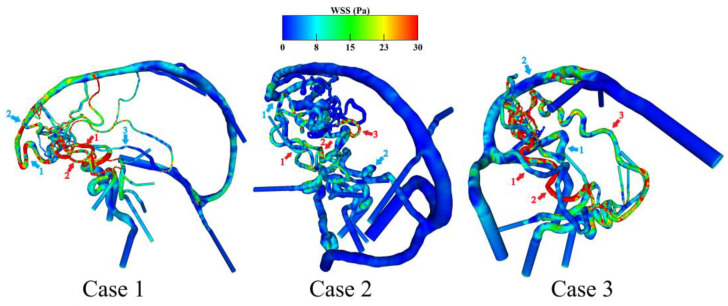
Qualitative representation of the WSS distribution on the luminal vessel wall. The red arrows including numbers indicate the feeding arteries, and the blue arrows including numbers show the draining veins, respectively. Local WSS elevations are particularly visible in the feeding arteries, which decrease in the direction of the distally located sinus veins.

Based on the generated surface models, subsequent investigations of the patient-specific flow behavior are achieved. Figure 6 shows the qualitative results of the calculated WSS on the luminal vessel walls for the AVM models.

It is visible that particularly in case 1, the two feeding arteries exhibit an increased WSS compared to arterial vessels located more proximal and in the posterior circulation. Furthermore, the frontally located draining veins in case 1 show increased WSS values when compared to cases 2 and 3. For case 2, the effect of increased feeding artery WSS is less prominent. Both the draining veins and the nidus itself exhibit the lowest shear stresses among the three cases. In contrast to the other two cases, the nidus of case 3 is mainly supplied by the posterior circulation. This is visible by the remarkably increased WSS over the whole feeding artery course of the right posterior cerebral artery in comparison to the other two feeding vessels coming from the anterior circulation.

Table 2 shows the quantification of the WSS in both the numbered feeding arteries and draining veins from Figure 6. The results support the qualitative observations. Case 2 exhibits the lowest WSS values across all the feeding arteries. Case 3 shows a remarkable increase in WSS on feeding artery 2, which canbe identified as the main branch supplying the nidus. Over all cases, the draining veins show reduced WSS values, which are most pronounced in case 2.

### 3.3. Analysis of the AVM-Related Blood-Drawing Effect

**Figure 7:**
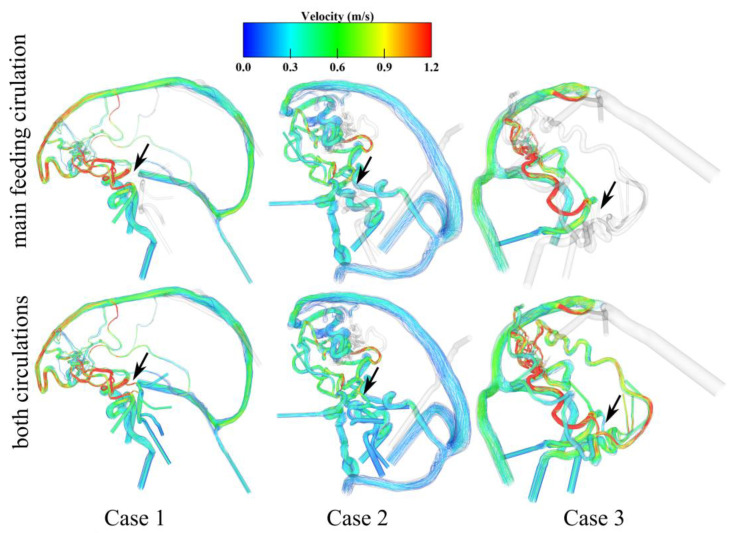
Qualitative representation of flow distribution using velocity-encoded streamlines. The first row demonstrates the flow distribution into the nidus only through the main feeding circulation of the Circle of Willis. The second row indicates changes in the posterior communicating artery's blood flow (see black arrows) having both circulations activated.

In addition to the shear-related behavior, flow distributions using velocity-encoded streamlines are presented in Figure 7. Here, particular focus relies on the flow behavior in the posterior communicating arteries, which connect the anterior and posterior blood flow supply. Therefore, the flow is visualized by the main feeding circulation (anterior for cases 1 and 2 and posterior for case 3, see upper row in Figure 7) and compared to the flow inside the posterior communicating arteries when looking at the flow emitted from the remaining circulation (see lower row in Figure 7). By comparing the results, it is notable that the flow is directed towards the dominant circulation by the AVM nidus. For cases 1 and 2, the flow gets distributed to the anterior side by the frontally located nidus. Case 3 is mainly supplied by the posterior circulation, resulting in a visible shunt effect of pulling the flow from the anterior side towards the posterior side and nidus.

This blood-drawing effect by the nidus is specified using the volume flow rate and direction quantifications in Table 3. It is visible for cases 1 and 2 that the volume flow rate is directed from the posterior towards the anterior circulation, as the nidus is located in the frontal part. In contrast, the negative values for case 3 indicate the reverse flow direction towards the posteriorly located nidus. Here, a distinct dominance of the flow rate on the right side with 255.5 mL/min is apparent due to the main supply from feeding arteries on the right side.

## 4. Discussion

The presented interdisciplinary approach allows the investigation of hemodynamic information inside patient-specific AVM models. For the three exemplary cases with high levels of complexity, a sophisticated workflow was developed to utilize a high amount of multimodal medical image data. The variety of input data serves as a base for the 3D model generation to achieve patient-specific AVM models with a detailed resolution. Based on these comprehensive surface meshes, image-based blood flow simulations allow the evaluation of the hemodynamic impact of the nidus on the surrounding vasculature.

Due to the multimodality of medical image data, large regions of interest could be extracted. Especially by using the MRA and MRV data, it was possible to extract the vessels of the whole Circle of Willis (including regions that were not feeding the AVM nidus) and the sinus vasculature (see Figure 5). The segmentation results allow the overall AVM morphology to be considered within a 3D context, showing almost all the nidus connections to the arterial and venous domains. This potential can be particularly useful in medical training and education. In comparison to recent AVM studies involving patient-specific image segmentation and blood flow simulations [14,15], the use of patient-specific QMRA blood flow measurements in this study increased the robustness of conducting realistic hemodynamic simulations. Since AVMs contribute considerably more to the redistribution of blood due to their shunt properties, such patient-specific in- and outflow rates are crucial for obtaining reliable results.

The hemodynamic results lead to the analysis of WSS and the flow velocity magnitude. In neurovascular research, the WSS is one of the most meaningful parameters regarding the cellular remodeling of pathological vessel walls and their rupture risk [23]. The simulation results show increased WSS at feeding arteries, meaning that there is a higher flow in these vessels (see Figure 6). In particular, for cases 1 and 3, the stress in the main feeding vessels is more pronounced compared to the large locally preceding arteries as well as the contralateral side. These qualitative results are in line with the investigations of Ye et al. [24]. In addition, Chang et al. [25] noticed such increased shear stresses in the feeding arteries of symptomatic AVMs. Furthermore, cases 1 and 3 indicate locally increased WSS at the draining veins, which might end up in a free flow effect at the draining vein to the sinus junction area. This is important since draining veins show higher volume flows which might play an important role in the formation of AVM-related side pathologies like venous stenoses [26] or venous varix [27]. Finally, the results of the velocity-encoded streamlines demonstrate the flow inside the nidus compartments as well as the steal phenomena (see Figure 7). The AVM nidus pulls the flow towards its draining veins from the Circle of Willis. Cases 1 and 2 lead the blood flow in the direction of the anterior circulation due to their frontally located nidus. However, in case 3, the occipitally located nidus causes a reversal of the flow direction in the posterior communicating arteries (see Table 3). Here, the dominant posterior side leads to the blood-steal effect in this direction.

Overall, as the first of its kind, this study presents a novel approach that integrates multiple imaging datasets to generate comprehensive, patient-specific 3D models with realistic blood flow behavior based on precise flow quantification. The proposed workflow enables the detailed and anatomically accurate representation of the AVM nidus, allowing for the distinction of compartments and vascular regions. Additionally, this method provides an in-depth hemodynamic characterization and quantification of critical vascular structures, including the entire course of feeding arteries and draining veins. Through this approach, previously unresolved insights into the complex AVM hemodynamics of these pathologies could be captured, which are not available with conventional imaging methods. This holds promising potential not only for enhancing the diagnosis and comprehension of the disease but also for assessing therapeutic approaches. Such hemodynamic models might be a base for the future computational modeling of intravascular treatment methods such as transarterial embolization [28] or novel transvenous techniques like the retrograde pressure cooker technique [29]. This can offer tremendous potential, particularly when choosing a suitable treatment option. During embolization, vessels that are under high hemodynamic stress can be precisely identified using the calculated WSS patterns and flow distributions. The area of supply in the nidus compartment of a respective feeder could be analyzed and the success of an embolization at this location could be estimated. In particular for staged embolization strategies, the appropriate order of the targeted vessels could be assessed to ensure treatment success while maintaining the hemodynamic balance or whether microsurgical intervention is required additionally.

Moreover, the usage of more advanced visualization in the neurosurgical domain using virtual and augmented reality has already demonstrated significant benefits, particularly in the visualization and surgical planning of brain tumors [30,31]. This enables neurosurgeons to overlay 3D segmentations of lesions onto real-world views of the patient’s brain, improving spatial awareness and precision during intervention. In AVM management, the precise visualization of the complex vascular anatomy is crucial for selecting optimal embolization strategies and determining the safest and most effective catheter pathways. The proposed advanced 3D image-based workflow can serve as a foundation for integrating augmented and virtual reality applications in clinical decision making and treatment planning.

Besides the presented advantages, there are several limitations. First, extensive manual segmentations of vessel structures from individual datasets were required. These had to be subsequently combined into an overall model and several artifacts were removed manually. This requires a large temporal effort and thus is not applicable in clinical practice yet. In addition, medical imaging may not allow the realistic imaging of the nidus, as some vessels are smaller than the minimum possible resolution of the imaging systems. Second, the hemodynamic simulations contain simplifications such as the assumption of rigid walls. Furthermore, for the AVM models, no specifications about the thickness and composition of the vessel walls were measured. However, there is promising progress in extracting vascular walls using neuro-optical coherence tomography. These recent advances are particularly successful in more distal arterial vessel areas and enable the volumetric imaging of the vessel walls [32]. Accordingly, upon obtaining reliable data regarding wall properties such as thickness and elasticity, the possibility of incorporating fluid–structure simulations may be explored. Third, although blood exhibits evident non-Newtonian characteristics, particularly in small vessels, it was treated as a Newtonian fluid. Nevertheless, various studies have determined that opting for Newtonian properties in the considered range of shear rates is a reasonable choice [33,34]. As further demonstrated in an uncertainty analysis conducted by Voß et al. [35], viscosity modeling has the smallest impact on variations in the simulation results. Furthermore, commonly applied shear-thinning models like Carreau-Yasuda have been shown to overpredict non-Newtonian effects in certain vascular regions [36]. Therefore, its influence on neurovascular AVM hemodynamics is expected to be minimal in the context of our study.

Finally, this study has a sample size of only three cases, which is primarily due to the complexity of acquiring multiple imaging modalities for each patient, including MR-based flow quantification. Furthermore, intracranial AVMs represent a relatively rare condition, limiting the availability of suitable cases based on this single-center approach. Nevertheless, the selected examples are chosen carefully to cover a range of anatomical variations, including both frontal and deep nidus localizations and different vessel diameters. Therefore, the limited number of cases is considered to be acceptable, as the primary goal of this exploratory study is to demonstrate the feasibility of the proposed workflow from a technical perspective. For future work, multi-center data acquisition could support the validation and generalization of the workflow across a broader patient population.

## 5. Conclusions

The proposed workflow study evaluates the feasibility of generating realistic AVM models and corresponding hemodynamic predictions based on a high variety of medical image data. These 4D elaborations, which are applied to three exemplary cases, can provide neurosurgeons and neuroradiologists with in-depth insights into challenging AVM pathologies. The hemodynamic results show the flow-related arterial and venous impact by using extensive 3D geometries and thus provide valuable guidance on the most suitable access pathways for the endovascular treatment of the AVM nidus. For example, these insights can be utilized in treatment planning to optimize catheter navigation, assess the feasibility of embolization strategies, and evaluate the potential hemodynamic consequences of different intervention approaches. Thus, the novelty of the detailed combination of the high multimodality in the image data and the interdisciplinarity of the workflow contributes substantially to the hemodynamic understanding of complex AVM flow phenomena.

## Figures and Tables

**Table 1:** Morphologic characteristics of the three AVM cases.

	Case 1	Case 2	Case 3
Nidus location	right frontal	right frontal	right occipital
Number of feeding arteries	2	3	3
Number of draining veins	3	2	2

**Table 2:** Quantitative results of the WSS on the luminal walls of the feeding arteries and draining veins of the three representative cases. In addition to the mean values for the respective vessel type, the relative deviation between feeding arteries and draining veins is indicated. Abbreviation: n/a = not available; rel. dev. = relative deviation.

	Case 1	Case 2	Case 3
WSS feeding artery 1 in Pa	52.6	7.7	5.1
WSS feeding artery 2 in Pa	17.0	7.1	46.2
WSS feeding artery 3 in Pa	n/a	12.8	12.5
Mean value (standard dev.) in Pa	34.8 (17.8)	9.2 (2.6)	21.3 (17.9)
WSS draining vein 1 in Pa	19.0	2.6	9.5
WSS draining vein 2 in Pa	15.5	3.3	9.5
WSS draining vein 3 in Pa	3.4	n/a	n/a
Mean value (standard dev.) in Pa	12.6 (6.7)	2.9 (0.3)	9.5 (0.01)
Rel. dev. of feeding arteries to draining veins	63.7%	68.2%	55.5%

**Table 3:** Volume flow rate of both posterior communicating arteries captured in the directions towards the anterior circulation. Note that for case 3 the flow values are negative, demonstrating the blood-drawing effect by the posteriorly located nidus.

	Case 1	Case 2	Case 3
RPcom volume flow rate in mL/min	70.8	122.4	−255.5
LPcom volume flow rate in mL/min	40.2	n/a	−40.8

## Data Availability

Data are available upon reasonable request.

## Abbreviations

The following abbreviations are used in this manuscript:

3DRA	3D rotational angiography
AVM	arteriovenous malformations
DICOM	Digital imaging and communications in medicine
CFD	computational fluid dynamics
LPcom	Left posterior communicating artery
MRA	magnetic resonance angiography
MRV	magnetic resonance venography
NOVA	non-invasive optimized vessel analysis
QMRA	phase-contrast quantitative magnetic resonance imaging
RPcom	Right posterior communicating artery
WSS	wall shear stress
